# ADVANCING GERONTOLOGICAL HEALTH EQUITY: INNOVATIONS AND INSIGHTS FROM HISPANIC SERVING INSTITUTIONS

**DOI:** 10.1093/geroni/igae098.1581

**Published:** 2024-12-31

**Authors:** Elizabeth Vasquez, Patricia Heyn

**Affiliations:** Chair; Discussant

## Abstract

In response to the Biden-Harris administration’s significant investment in Hispanic Serving Institutions (HSIs), this symposium showcases groundbreaking research and projects that address critical public health and gerontological issues within these communities. This symposium highlights four innovative, multidisciplinary approaches that demonstrate the effectiveness of community-engagement research, clinical studies, and health promotion in addressing the needs of older adults. Firstly, Dr. Jung-Ah Lee’s presentation delves into community engagement projects led by Latino nursing students and faculty at the University of California, Irvine. These projects emphasize compassionate, hands-on initiatives aimed at improving the health and well-being of Latino older adults in Southern California, including in-home caregiving education and support for caregivers of people living with dementia. Next, ERASE Falls project, spearheaded by Dr. Pappa at Marymount University, exemplifies the expansion and scalability of evidence-based fall prevention programs. This initiative has significantly impacted Northern Virginia’s diverse older adult population, demonstrating the power of community partnerships and innovative training models to enhance fall prevention efforts. Next, Dr. Suriaga’s research provides critical insights into the association between drug intoxication and the co-use of opioids, cannabis, and alcohol among Hispanics in Florida. This work highlights the urgent need for targeted research to understand and address substance use and its effects within Latino communities. Lastly, Alcantar work underscore the essential role of HSIs in fostering gerontological health equity through research, education, and community engagement. Attendees will gain a comprehensive understanding of challenges and opportunities in gerontological research conducted by HSIs researchers and the importance of culturally sensitive approaches. Hispanic Serving Institutions Interest Group Sponsored Symposium

